# Advanced Algorithms for Local Routing Strategy on Complex Networks

**DOI:** 10.1371/journal.pone.0156756

**Published:** 2016-07-19

**Authors:** Benchuan Lin, Bokui Chen, Yachun Gao, Chi K. Tse, Chuanfei Dong, Lixin Miao, Binghong Wang

**Affiliations:** 1 Department of Modern Physics, University of Science and Technology of China, Hefei, China; 2 School of Computing, National University of Singapore, Singapore, Singapore; 3 Faculty of Information Technology, Macau University of Science and Technology, Macau, China; 4 School of Physical Electronics, University of Electronic Science and Technology of China, Chengdu, China, Chengdu, China; 5 Department of Electronic and Information Engineering, Hong Kong Polytechnic University, Hung Hom, Kowloon, Hong Kong; 6 Division of Logistics and Transportation, Graduate School at Shenzhen, Tsinghua University, Shenzhen, China; 7 Center of Environmental Science and New Energy Technology, Tsinghua-Berkeley Shenzhen Institute, Shenzhen, China; 8 Department of Atmospheric, Oceanic and Space Science, University of Michigan, Ann Arbor, United States of America; East China University of Science and Technology, CHINA

## Abstract

Despite the significant improvement on network performance provided by global routing strategies, their applications are still limited to small-scale networks, due to the need for acquiring global information of the network which grows and changes rapidly with time. Local routing strategies, however, need much less local information, though their transmission efficiency and network capacity are much lower than that of global routing strategies. In view of this, three algorithms are proposed and a thorough investigation is conducted in this paper. These algorithms include a node duplication avoidance algorithm, a next-nearest-neighbor algorithm and a restrictive queue length algorithm. After applying them to typical local routing strategies, the critical generation rate of information packets *R*_*c*_ increases by over ten-fold and the average transmission time 〈*T*〉 decreases by 70–90 percent, both of which are key physical quantities to assess the efficiency of routing strategies on complex networks. More importantly, in comparison with global routing strategies, the improved local routing strategies can yield better network performance under certain circumstances. This is a revolutionary leap for communication networks, because local routing strategy enjoys great superiority over global routing strategy not only in terms of the reduction of computational expense, but also in terms of the flexibility of implementation, especially for large-scale networks.

## Introduction

Communication networks play increasingly integral roles in our’s daily lives. In recent years, a great of research effects have been devoted to improving the performance and efficiency of communication networks. Scientists have analyzed the problem in two main aspects: network structure [1, 2] and routing strategies [3–10]. Owing to the high cost of modifying network structure, most studies have resorted to improving routing strategies to achieve better performance at reasonable cost of implementation. Routing strategies on communication networks can be divided into two categories: one is the local routing strategy, including local static routing [3], local dynamic routing [4] and local pheromone routing strategy [5]; the other is the global routing strategy, such as shortest path routing strategy [6–8], efficient routing strategy [9] and global dynamic routing strategy [10]. The difference between global and local routing strategies is that a global routing strategy requires global information, including the topological structure of the whole network, the characteristics of each node or the real-time information of each transmitted packet. In a local routing strategy, however, each node only needs to acquire some local information, involving only its neighbors or next-nearest-neighbor for example, without the need to know any global information at any time. Therefore, local routing strategies remain highly promising for practical implementation on account of the ever-increasing cost both in labor and time to grasp the complete information of the networks whose nodes are growing exponentially with time in reality. Similar to routing strategy in communication networks, in traffic networks, route guidance strategy is utilized to calculate and define the route of vehicles. In order to reduce the cost, the studies in traffic science also focus on the local route guidance strategies [11–16].

In spite of the low cost and the ease of implementation in large-scale networks, the transmission efficiency of local routing strategies is much lower than that of global routing strategies. In this paper, we analyze the cause of the poor efficiency of local routing strategies. This leads to three special algorithms that can significantly improve the transmission efficiency and outperform global routing strategies in some cases. The paper is organized as follows. In Section 2, the definition of the scale-free network and the existing routing strategies are introduced. In Section 3, the reasons for low transmission efficiency of the local pheromone routing strategy are analyzed. Prompted by this analysis, we propose three algorithms and then apply them to the local pheromone routing strategy. Moreover, three algorithms are also applied to other local routing strategies in Section 4. In Section 5, the global routing strategies and our improved local routing strategies are compared in detail. Finally, Section 6 concludes the paper.

## Models and Routing strategies

### BA model

Previous studies have indicated that many real-world networks follow a power-law degree distribution. Among all the models, the BA network [17], proposed by Barabási and Albert in 1999, is one of the closest to a real-world network. It is constructed using the following procedures:

1. It starts from *m*_0_ fully connected vertices, where *m*_0_ is a small number.
2. At each time step, a new vertex with *m* (≤*m*_0_) edges linked to *m* various vertices is added to the existing graph.
3. The addition has a preferential attachment, i.e., the probability that the new vertex is connected to the existing node *i* is proportional to the degree *k*_*i*_ of node *i*,
  $$\begin{array}{c} P\left(k_{i}\right)=\frac{k_{i}}{\sum_{j}k_{j}}. \end{array}$$ (1)

After *t* time steps, the total number of vertices is *N* = *t* + *m*_0_, while the number of the incremental edges is *mt* and the network evolves into a scale-free state with a power-law degree distribution *p*(*k*)∼*k*^−3^ when *t* is large enough.

### Critical generation rate

The critical generation rate of information packets and the average transmission time are two key physical quantities for assessing the efficiency of routing strategies on complex networks. For simplicity, we assume that all nodes act as both hosts and routers and have the same capabilities in delivering information packets, which means that each node can deliver at most *C* packets per time step. At each time step, there are *R* information packets generated in the network with randomly chosen origins and destinations [18]. These packets are delivered according to the rules of the routing strategy used and will be removed upon reaching the destinations. If *R* is fairly small, the numbers of created and removed information packets are balanced, leading to a steady state known as the free-flow state. On the contrary, if *R* is pretty large, the number of created packets will exceed that of the removed ones, which will give rise to a continued accumulation of the packets and eventually lead to complete congestion. Therefore, as the value of *R* increases, the network experiences a phase transition from a free-flow state to a congestion state. The value at the phase transition point is the critical generation rate of information packets, denoted as *R*_*c*_. We use the order parameter *η* to characterize the phase transition [19]:

$$\begin{array}{c} \eta \left(R\right)=\lim_{t\to \infty }\frac{C}{R}\frac{\left\langle \Delta N_{p}\right\rangle }{\Delta t}, \end{array}$$ (2)

where Δ*N*_*p*_ = *N*_*p*_(*t* + Δ*t*) − *N*_*p*_(*t*) denotes the change of the total number of information packets within a time interval Δ*t*. This order parameter indicates the ratio between the outflow and the inflow of information packets. For *R* < *R*_*c*_, 〈Δ*N*_*p*_〉 → 0 and *η* → 0, indicating that the system is in a free-flow state. While for *R* > *R*_*c*_, only a few packages could be transmitted to destination. So, *N*_*p*_(*t* + Δ*t*) − *N*_*p*_(*t*) ≈ *R*Δ*t* and *η*(*R*) → *C*, indicating that information packets are continually accumulated and the system will collapse ultimately. Hence, *R* = *R*_*c*_ represents the phase transition point.

### Routing strategies

#### Local routing strategies

The first local routing strategy we introduce is called local static routing strategy [3]. It proceeds as follows: all nodes perform a parallel local search among their immediate neighbors. If a packet’s destination is found within the searched area, it will be delivered directly to the destination; otherwise, it will be forwarded at the probability according to the neighbors’ degrees:

$$\begin{array}{c} \Pi_{i\to j}=\frac{k_{j}^{\alpha }}{\sum_{r}k_{r}^{\alpha }}, \end{array}$$ (3)

where Π_*i* → *j*_ is the probability of the information packets transmitted from node *i* to neighbor node *j*. *k*_*r*_ is the degree of node *r* which is the neighbor of node *i*. *α* is a tunable parameter. It has been proved that when *α* = −1, the strategy has the best performance of information packets delivery [3]. That means a node with a small degree enjoys a high forwarded probability. The characteristics of this strategy are low-cost and the impressive transmission efficiency compared with the random-walking routing strategy.

Subsequently, Wang *et al*. [4]. proposed the local dynamic routing strategy, in which the dynamic information of the queue lengths of neighbors is taken into consideration, i.e.,

$$\begin{array}{c} \Pi_{i\to j}=\frac{k_{j}{\left(n_{j}+1\right)}^{\beta }}{\sum_{r}k_{r}{\left(n_{r}+1\right)}^{\beta }}, \end{array}$$ (4)

where Π_*i* → *j*_ is the probability of the information packets transmitted from node *i* to neighbor node *j*. *k*_*r*_ is the degree of node *r* which is the neighbor of node *i*. *n*_*r*_ is the number of packets in the queue of node *r*. *β* is a tunable parameter. The neighbor with larger *n*_*i*_ is more probable to be delivered if *β* > 0, and the opposite is true if *β* < 0. The best performance emerges at *β* = −3 [4].

Furthermore, Ling *et al*. [5] proposed the local pheromone routing strategy, enlightened by a chemical substance, pheromone [20], which is laid by ants so that they can pick up their trails. To mimic the production and evaporation of the pheromone to iterate to the best route, the algorithm defines:

$$\begin{array}{c} \Pi_{i\to j}=\frac{p_{ij}^{\alpha }}{\sum_{r}p_{ir}^{\alpha }}, \end{array}$$ (5)

where Π_*i* → *j*_ is the probability of the information packets transmitted from node *i* to neighbor node *j*. *p*_*ij*_ is the pheromone of the link pointing from node *i* to *j*, and *α* is a tunable parameter. Initially, the value of the pheromone concentration on each link is small (set to be 0.001). Let *L*_*C*_ be the critical queue length of a node. When a packet is delivered successfully from node *i* to node *j*, the pheromone of link decreases by a unit if *L*_*j*_ > *L*_*C*_, i.e.,

$$\begin{array}{c} p_{ij}=\max\left\{p_{ij}-\delta p,\;\delta p\right\}. \end{array}$$ (6)

Otherwise, if *L*_*j*_ ≤ *L*_*C*_, the pheromone of link increases by a unit, i.e.,

$$\begin{array}{c} p_{ij}=p_{ij}+\delta p \end{array}$$ (7)

*L*_*C*_ is set as *L*_*C*_ = *βC* and *β* is a tunable parameter. The best performance emerges at *α* = 1 and *β* = 2 [5].

#### Global routing strategies

The first widely studied global routing strategy is the shortest path routing strategy, in which the information packets are delivered in the shortest path from origin to destination. However, the critical generation rate is very low and nodes with high degrees are blocked as well. In 2006, Yan *et al*. [9] proposed a new global routing strategy, named efficient routing strategy. In this strategy, the path *P*_*i* → *j*_ between nodes *i* (origin) and *j* (destination) is defined as follows:

$$\begin{array}{c} P_{i\to j}=\min\left\{\sum_{l=0}^{N_{L}}k{\left(x_{l}\right)}^{\beta }\right\}, \end{array}$$ (8)

where *x*_*l*_ represents the nodes on the path between nodes *i* and *j*, and *N*_*L*_ is the number of the nodes on the path between nodes *i* and *j*. *k*(*x*_*l*_) is the degree of node *x*_*l*_ and *β* is a tunable parameter. The best performance appeared when the tunable parameter *β* = 1, which corresponds to the path that has the minimal value of the sum of node degrees.

Based on this, the global dynamic routing strategy was enhanced later on by Ling *et al*. [10], who suggested that the transfer path *P*_*i* → *j*_ should be the minimal sum queue length of nodes:

$$\begin{array}{c} P_{i\to j}=\min\left\{\sum_{l=0}^{N_{L}}\left[1+n\left(x_{l}\right)\right]\right\} \end{array}$$ (9)

where *x*_*l*_ represents the nodes on the path between nodes *i* and *j*, and *N*_*L*_ is the number of the nodes on the path between nodes *i* and *j*. *n*(*x*_*l*_) represents the real-time queue length of information packets of node *x*_*l*_.

Although simulation results indicate that global routing strategy have pervasively larger critical value *R*_*c*_ than local routing strategies, they are expensive and impractical for large scale networks. In this light, we will focus on local routing strategies, aiming to improve their performance.

## Results and Discussion

In the following simulation, the BA network parameters are fixed. The network size is *N* = 1024, the mean degree is *K* = 10 and the delivering capability of each node is *C* = 5.

**Fig 1:**
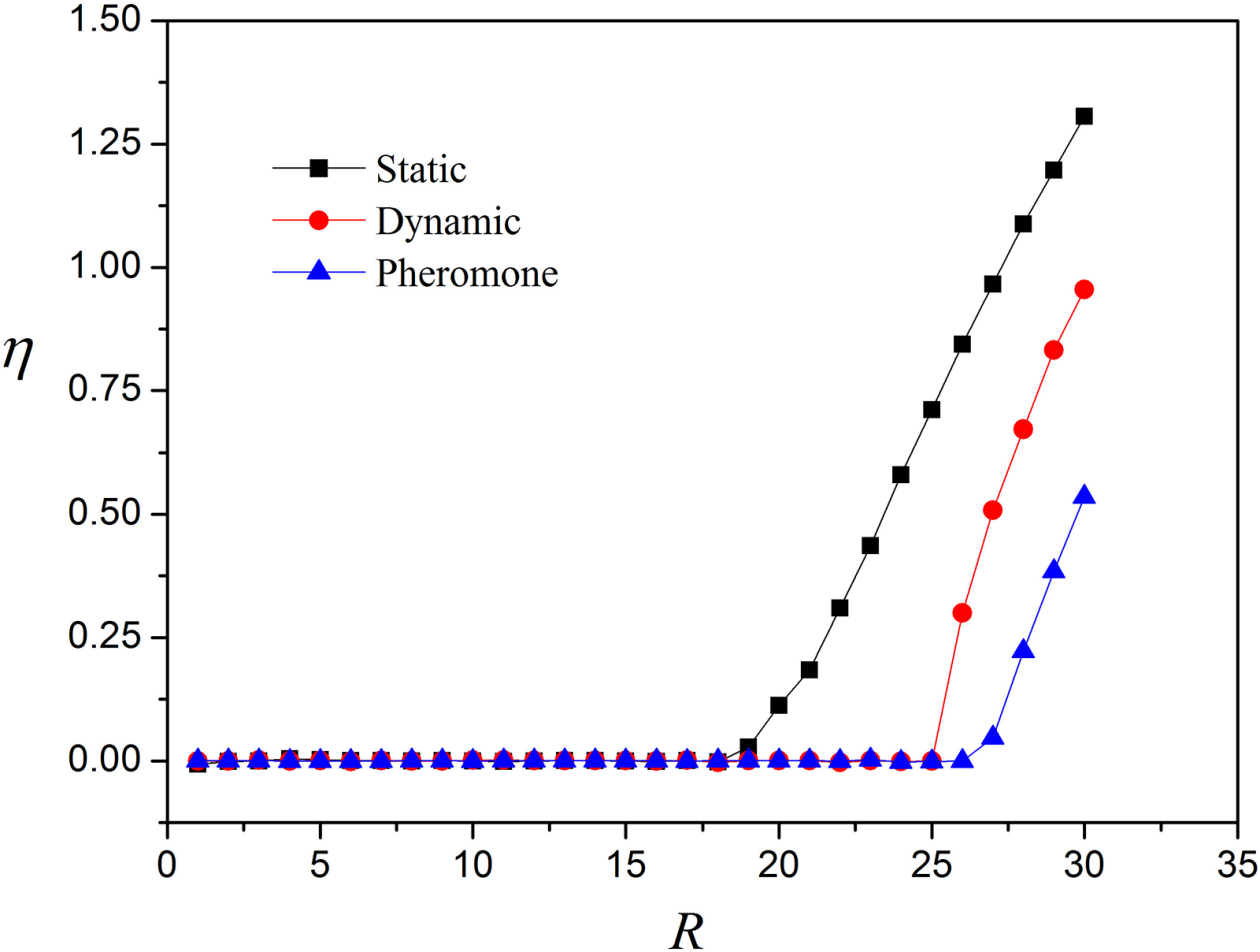
Order parameter *η* versus *R* for different routing strategies. Local static routing strategy, local dynamic routing strategy and local pheromone routing strategy.

Fig 1 shows the order parameter *η* versus *R* for several local routing strategies, in which local static routing strategy has the poorest performance (*R*_*c*_ = 18). The reason is that the dynamic information taken into account in the other two strategies is ignored in the local static case. Furthermore, the local pheromone routing strategy has the largest critical value *R*_*c*_ = 26. Thus we will apply our enhanced algorithms to this local routing strategy.

### Node duplication avoidance algorithm

**Fig 2:**
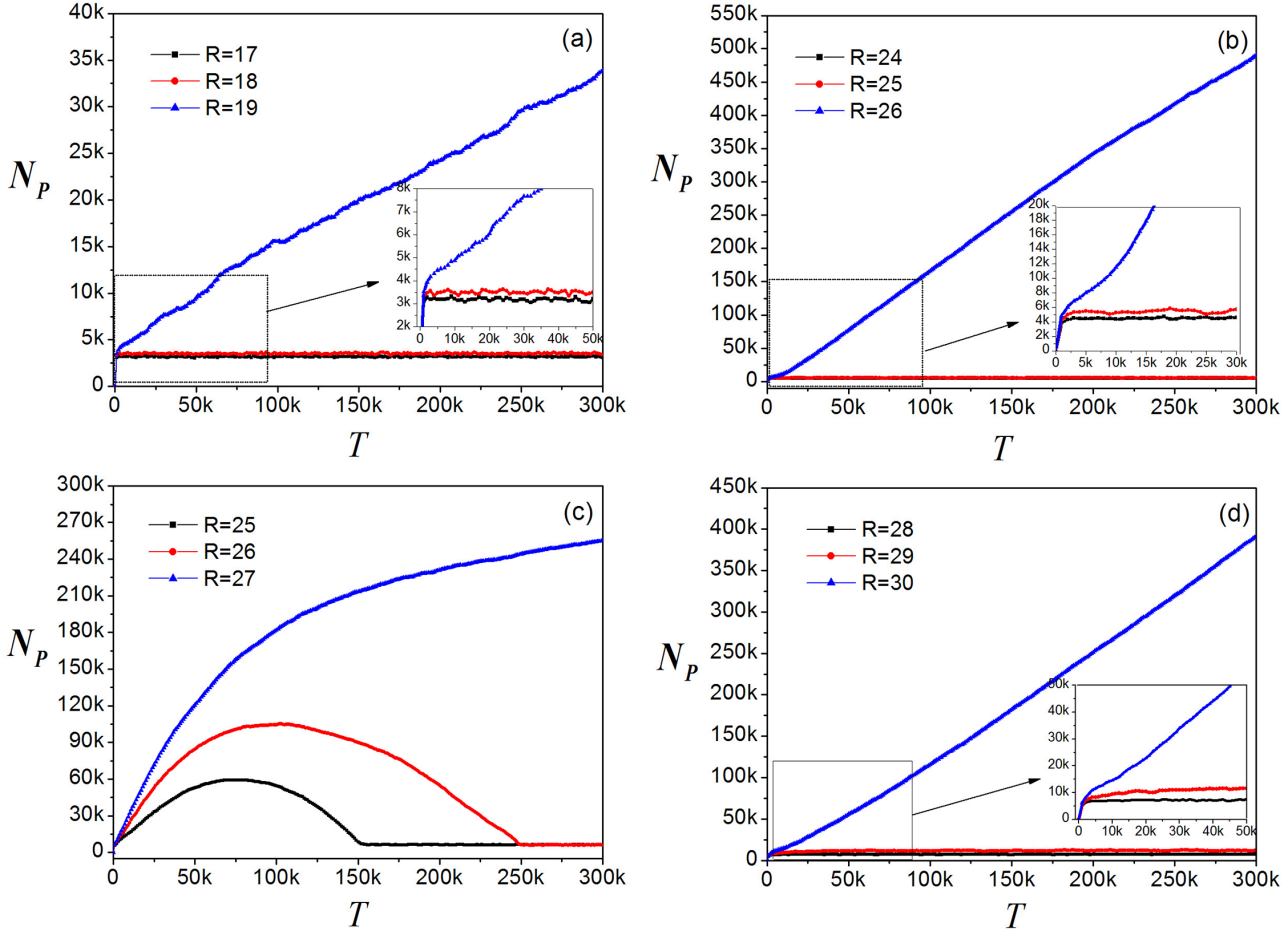
Evolutions of information packets. (a) local static routing strategy; (b) local dynamic routing strategy; (c) local pheromone routing strategy; (d) NDA-pheromone routing strategy.

Unlike other local routing strategies, if information packets are transmitted in accordance with the local pheromone routing strategy, a relaxation stage appears when *R* is close to *R*_*c*_, as shown in Fig 2(a)–2(c). Although the local pheromone routing strategy brings a large *R*_*c*_ = 26, it takes nearly 10^5^ time steps to get into the free-flow state. That means an enormous amount of time is consumed in the initial period. This cost is related to the algorithm which chooses the successive node according to the pheromone concentration of the edges connected to it. Because the pheromone concentration of each edge is approximately identical in the initial period, there is a random walk tending to revisit nodes after a brief period of exploring fresh nodes. To put it in another way, 10^5^ time steps are the time needed to dynamically add the pheromone. In this light, we propose the node duplication avoidance algorithm (NDA):

1. Record the latest *W* nodes that the information packet has visited so that the packet would not be passed on to these nodes in the next transmission. In the unrecorded neighbors, information packets choose their next propagation node depending on the local pheromone routing strategy.
2. If all the neighbors are recorded, the next propagation node will still be chosen according to the local pheromone routing strategy.

**Fig 3:**
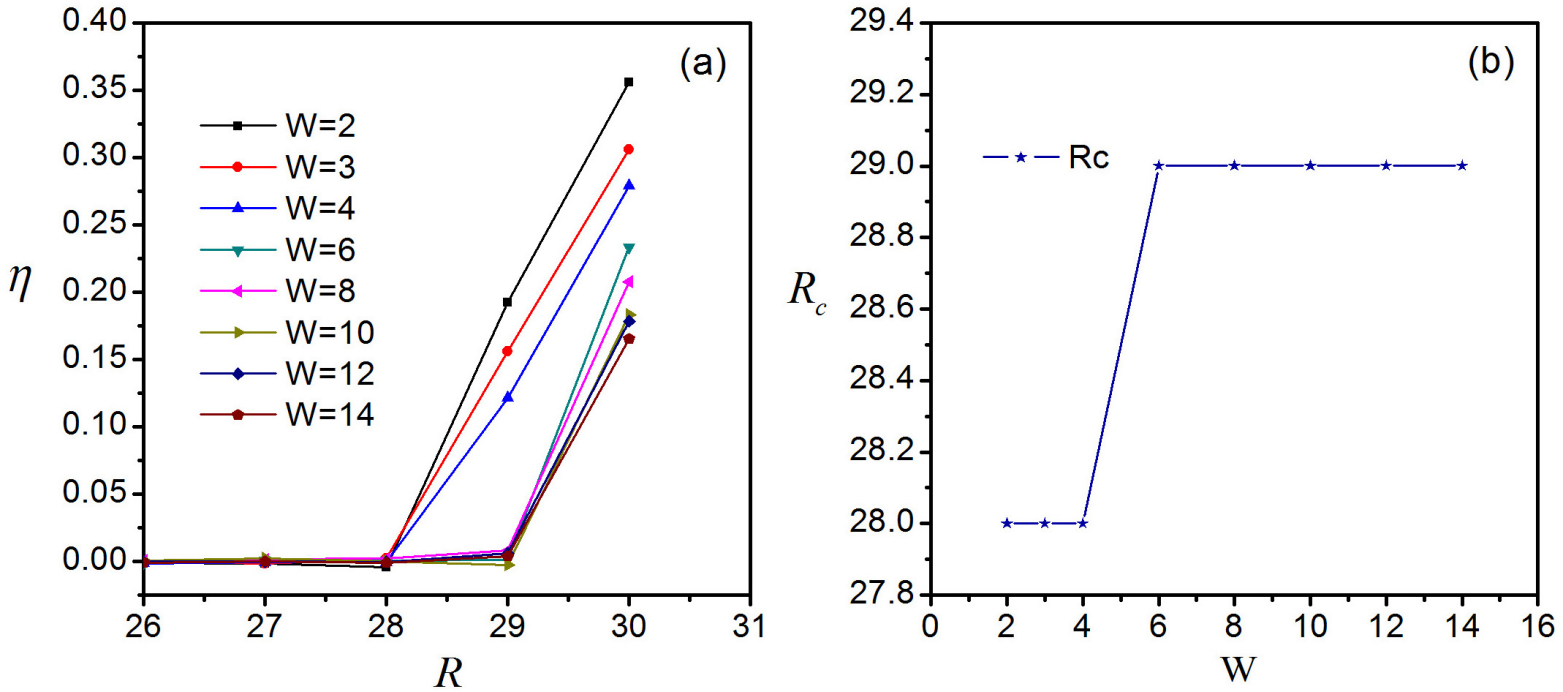
Influence of *W* in NDA algorithm. (a) Variance of *η* with increasing *R*; (b) variance of *R*_*c*_ with increasing *W*.

Apply the node duplication avoidance algorithm to local pheromone routing strategy, we obtain the new strategy named NDA-local pheromone routing strategy and called NDA-pheromone for short in figure. This algorithm is aimed to avoid circulation of packets in the process of transmission. We find the parameter *R*_*c*_ is influenced by the value of *W*. Fig 3(a) illustrates the change of the order parameter *η* versus *R* for different values of *W* and the relation between *R*_*c*_ and *W* is shown in Fig 3(b). It can be see that if *W* < 6, *R*_*c*_ = 28, and if *W* ≥ 6, *R*_*c*_ = 29, having increased by two and three units compared to the original *R*_*c*_ = 26, respectively. Thus *W* is set to 6 to reduce time consumption. The most important feature is that, in Fig 2(d) where the total packet number *N*_*p*_ versus time step *T* is depicted with *R* = *R*_*c*_ = 29, the fluctuation shown in Fig 2(c) disappears. This shows that our algorithm can improve *R*_*c*_ and avoid the circuitous transmission. Nevertheless, the improvement of the network capacity is very limited as *R*_*c*_ increases only from 26 to 29. We construct the following algorithms to provide further enhancement.

### Next-Nearest-Neighbor algorithm

**Fig 4:**
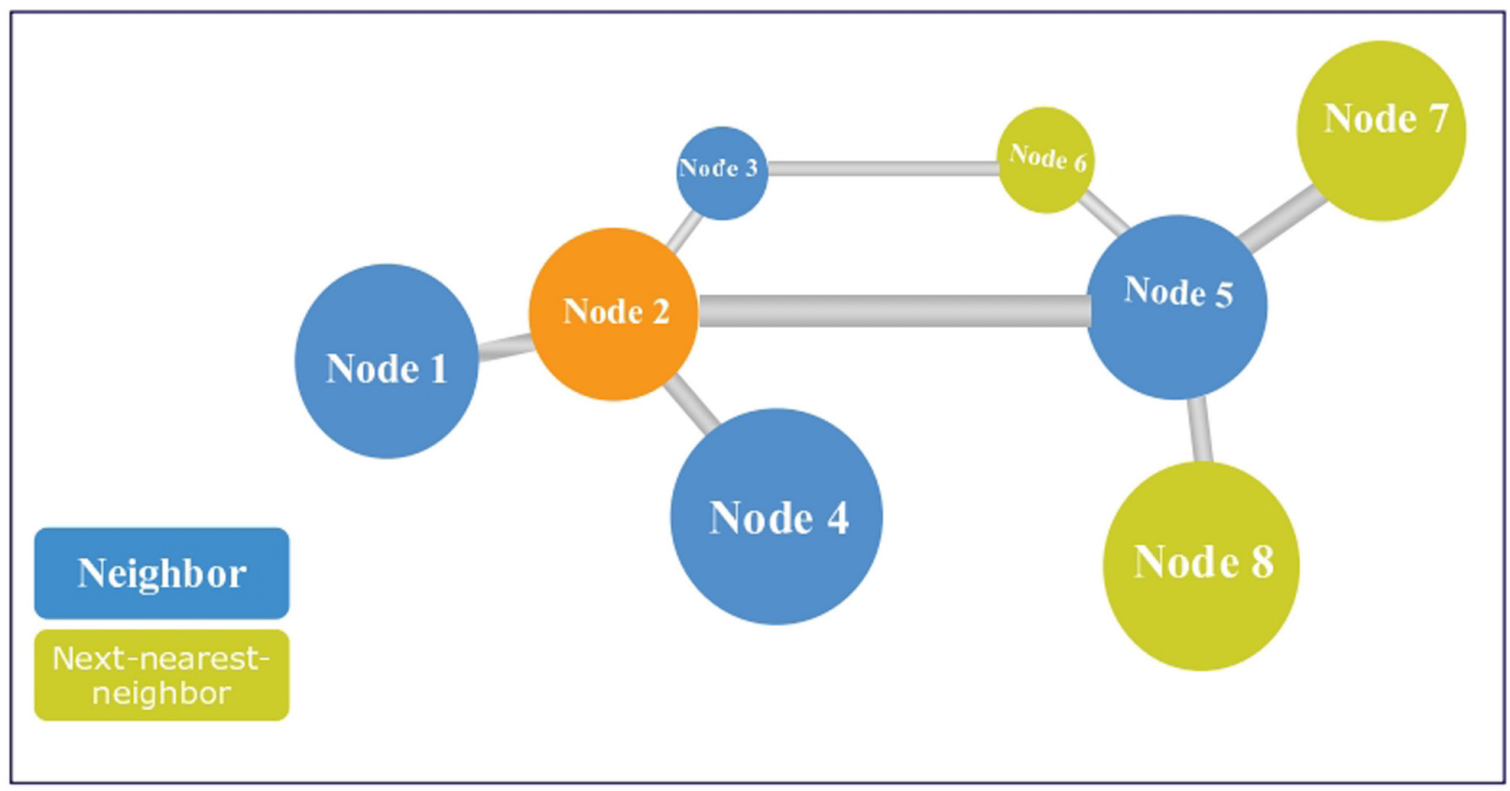
Illustration of next-nearest-neighbor node.

The concept of next-nearest-neighbor was presented by Adamic [18] in 2001. It is assumed that a node can obtain the information not only of its neighbors, but also of its neighbors’ neighbors, similar to a person having some knowledge of his or her friends’ friends. The next-nearest-neighbor is defined as the neighbors of a node’s neighbors. In Fig 4, for instance, the lime green nodes are the orange node’s next-nearest-neighbors. Here we apply this concept to the node duplication avoidance algorithm and propose the next-nearest-neighbor algorithm (NNN), which is stated as follows:

1. When an information packet arrives at a node, it first searches node’s neighbors to find the destination. If there exists a destination node, the packet will be delivered to it.
2. Otherwise, if the destination is one of the node’s next-nearest-neighbors, it will be delivered to the neighbor linked with the destination.
3. If the packet fails to transmit after the first two steps, the information packets will be propagated acting on NDA-local pheromone routing strategy.

**Fig 5:**
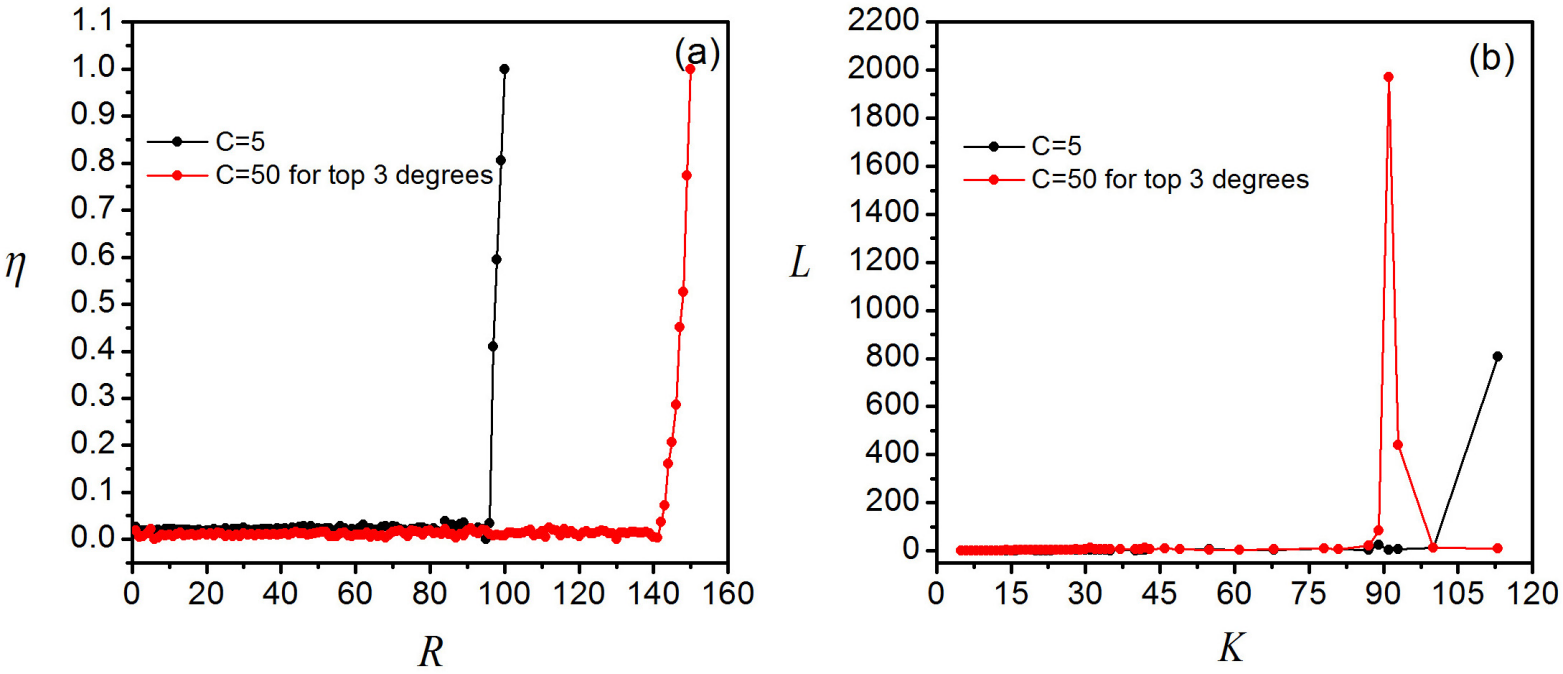
Simulation results of the Next-Nearest-Neighbor algorithm. (a) Order parameter *η* versus *R* for NNN-local pheromone routing strategy, *η* is normalized. (b) Queue length distribution with *R* > *R*_*c*_.

When the next-nearest-neighbor algorithm is applied to local pheromone routing strategy, the new strategy is obtained entitled as NNN-local pheromone routing strategy and called NNN-pheromone for short in figure. Similarly, the value of *R*_*c*_ is measured by plotting the order parameter *η* versus *R*, as shown by a black curve in Fig 5(a). From this figure, one can see that *R*_*c*_ = 96 about three times higher than the previous *R*_*c*_ = 29, demonstrating the effectiveness in increasing *R*_*c*_ by enlarging the search scope. In principle, the expanded search scope can decrease the time of a packet being stranded in a network caused by the unnecessary roundabout routes. In the simulation, however, we also find that the traffic jam would more easily occur in the large degree nodes. The curve in black in Fig 5(b) demonstrates the queue length distribution as *R* = 100 > *R*_*c*_. Only those nodes with high degrees would have enormous queue lengths while others are running well without congestion. The reason is that in the next-nearest-neighbor algorithm, the last two nodes in the delivery path is the neighbor and its attached destination node (i.e., the next-nearest neighbor). As the destination is randomly chosen, the node with large degree is more probable to be the preceding node of the destination (i.e., the neighbor). So the larger degree the node owns, the more packets it will receive and deliver to the destination eventually. This is the reason why congestion occurs most likely on hub nodes.

A simple way to alleviate this congestion is to enhance the delivering capability of the hub nodes. As shown by the red curve in Fig 5(a), *R*_*c*_ increases from 96 to 141 when the delivering capability of the node with the top three degrees is increased from 5 to 50. The red curve in Fig 5(b) shows that the queue lengths of the top three highest degrees nodes show a sharp decline. Network congestion, however, is still caused by the load of the nodes with the fourth largest degree when *R* > *R*_*c*_, revealing that the overloading pressure cannot be eliminated by enhancing the transmission capacity of the hub nodes. Therefore, we incorporate the following algorithm to further enhance the network capacity.

### Restrictive queue length algorithm

Although the transmission capacity of the network can be increased by using the next-nearest-neighbor algorithm, the problem of hub nodes handling excessive loads of transmitting packets remains unsolved. To ensure a more even distribution traffic loads for all nodes, we propose a restrictive queue length algorithm (RQL):

1. If the destination is found in the neighbors, the information packet will be delivered to it.
2. Otherwise, we set a threshold *s* + *t*. If a neighbor’s queue length does not exceed this value, the next-nearest-neighbors will be searched. Once a destination is found, the information packet is delivered to the neighbor linked with the destination.
3. We set a threshold *s*. Among the neighbors with the queue length being not more than *s*, the information packet is delivered in accordance with the first step of the NDA-local pheromone routing strategy.
4. If the packet fails to transmit after the previous steps, it will be delivered according to the NDA-local pheromone routing strategy.

**Fig 6:**
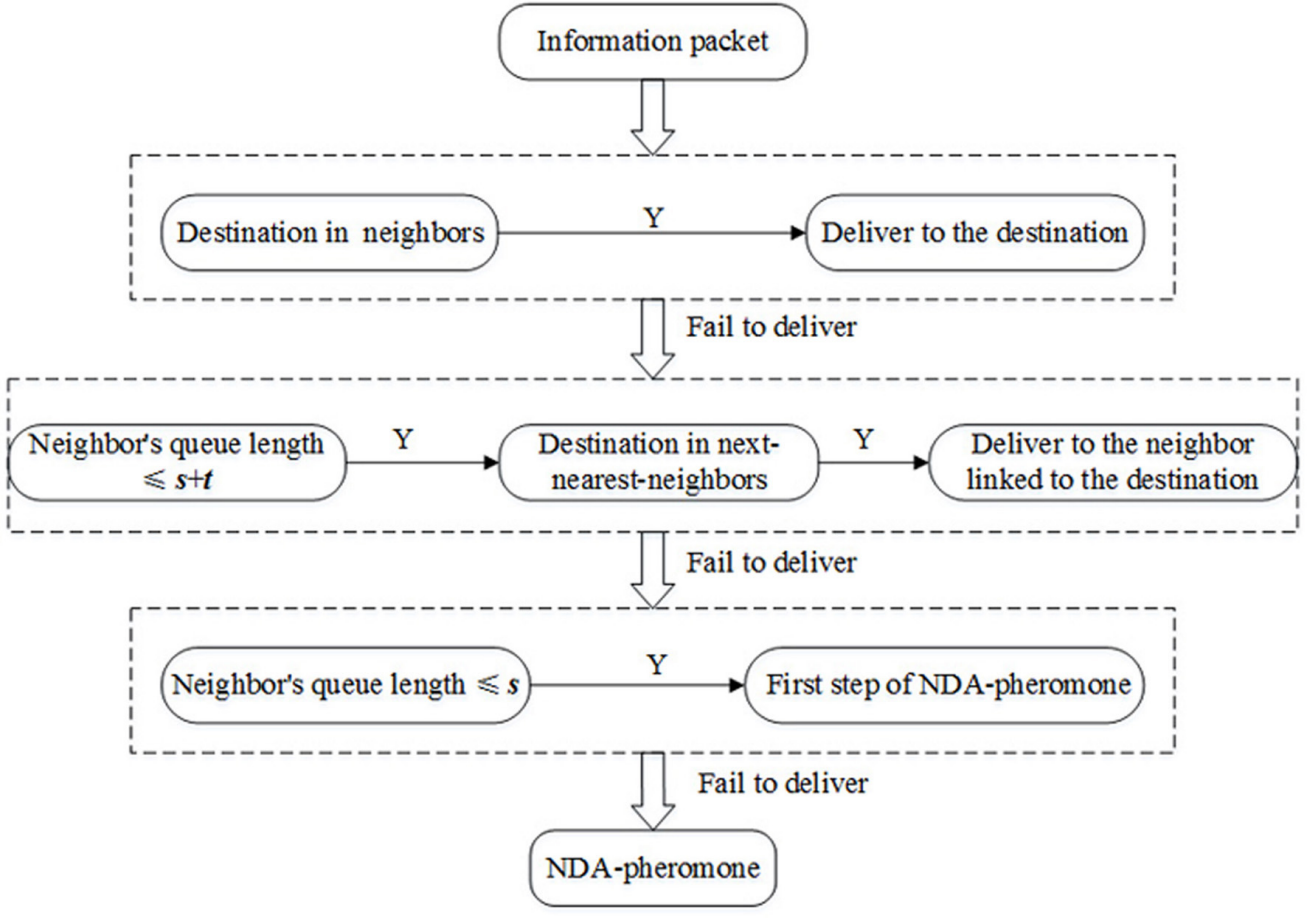
Framework of the restrictive queue length algorithm.

When the restrictive queue length algorithm is applied to local pheromone routing strategy, the new strategy is obtained entitled as RQL-local pheromone routing strategy and called RQL-pheromone for short in figure. Fig 6 shows the framework of the restrictive queue length algorithm. In comparison with the next-nearest-neighbor algorithm, this algorithm imposes the restriction of the package queue length. The information packet cannot be passed on to a busy node with a queue length exceeding the threshold. Thus as the existing queue length of a busy node decreases while it processes the packets, the queue length will fall below the threshold with time and then the node will be available to accept new information packets again. Considering that the next-nearest-neighbor algorithm can save the time wasted in roundabout paths, we set the threshold in the second step larger than that in the third step. In fact, the effect of roundabout paths can be analyzed from two aspects. If the queue length is not too long, it is worthy of waiting as the destination is found among the next nearest neighbors, because it only takes two steps in addition to the waiting time steps to reach the destination. However, if the queue length is so long that the waiting time takes most of the transmission time, then it may be better to go through a roundabout path to get to the destination. Thus the set threshold can converge to a finite optimal number.

**Fig 7:**
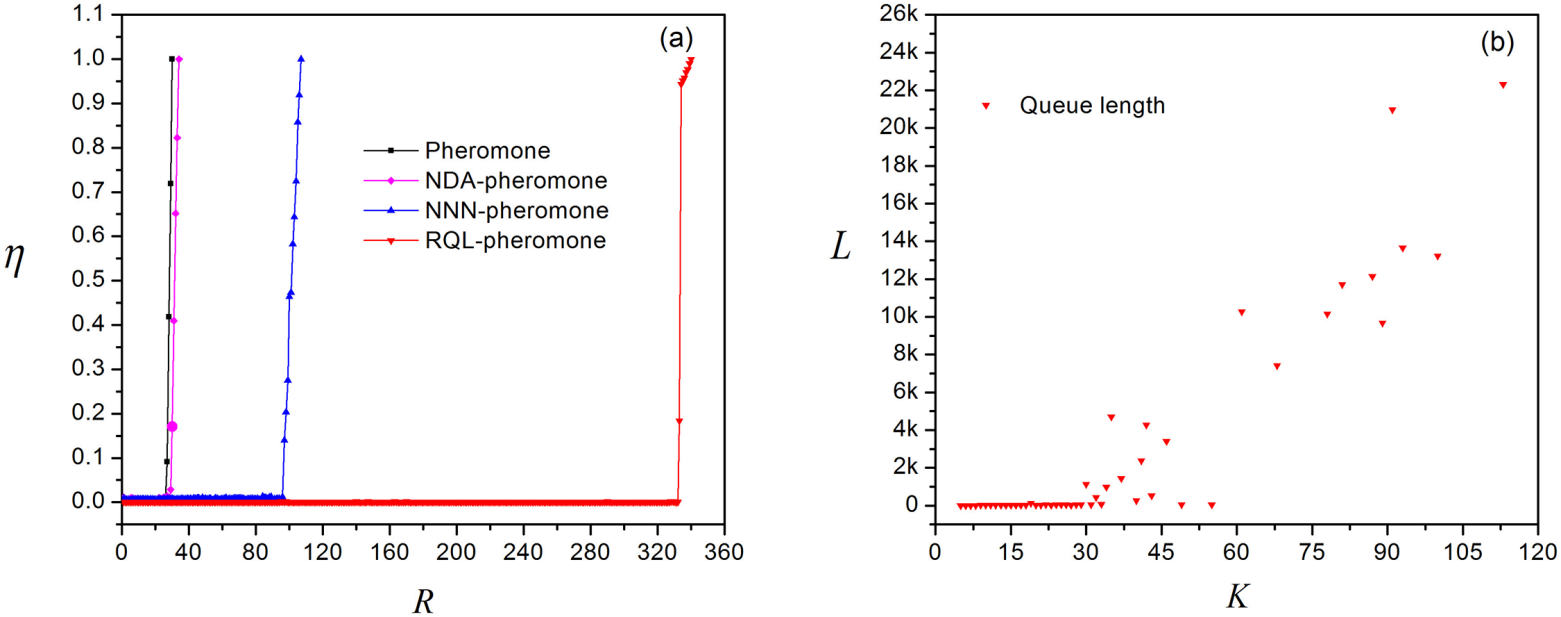
Simulation results of the Restricting Queue Length Algorithm. (a)Order parameter *η* versus *R* for four routing strategies with *η* is normalized. (b) Queue length versus node degree for *R* > *R*_*c*_.

The values of *s* and *t* can affect the transmission efficiency of the network. As *s* + *t* is a large value, an overwhelming majority of nodes meet one of the first three rules, thus the restrictive queue length algorithm is reduced to the next-nearest-neighbor algorithm. As *s* + *t* is a small value, a vast majority of node queue lengths are larger than the threshold and information packets are passed by the fourth step, thus the restrictive queue length algorithm is similar to the node duplication avoidance algorithm. These are also demonstrated in Table 1 which shows the critical generation rate of information packets for different pairs of *s* and *t*. When *s* + *t* ≥ 10000, *R*_*c*_ = 96, which is equivalent to the value of NNN-local pheromone routing strategy. In relative terms, when *s* = *t* = 0, *R*_*c*_ = 23 which is less than that of the node duplication avoidance algorithm. The reason for this is that the nodes meeting the second and third rules are only unoccupied nodes which, relatively speaking, are in possession of a smaller degree. If an information package is transmitted to a small degree node, it will be routed to the destination with difficulty. When 5 ≤ *t* ≤ 1000 and 10 ≤ *s* ≤ 1000, *R*_*c*_ can be over 300. The maximum value is 332 as *t* = 80, *s* = 20 (shown in Fig 7(a)). This means the critical generation rate of information packets of RQL-local pheromone routing strategy is 13 times higher than that of the local pheromone routing strategy approximately. This is the first and the most important advantage.

**Table 1:** The critical generation rate of information packets for different *t*, *s*.

	*s* = 0	*s* = 5	*s* = 10	*s* = 50	*s* = 100	*s* = 500	*s* = 1000	*s* = 5000	*s* = 10000
*t* = 0	23	181	212	311	314	318	320	142	96
*t* = 5	136	297	315	324	327	330	330	142	96
*t* = 10	198	304	317	326	329	331	331	143	96
*t* = 50	257	311	323	330	330	331	332	143	97
*t* = 100	261	314	325	331	331	331	331	142	96
*t* = 500	263	316	330	331	331	331	331	142	96
*t* = 1000	265	316	326	331	331	331	331	142	96
*t* = 5000	96	96	130	129	129	129	129	96	96
*t* = 10000	96	96	96	96	96	96	96	96	96

The second advantage of restrictive queue length algorithm is that it can disperse the traffic loads. To illustrate this point, we set the information generation rate *R* = 336 (>*R*_*c*_ = 332) and plot the queue length of nodes against the node degree. Fig 7(b) shows that the queue length is approximately linear to the degree. Therefore, compared to the former algorithm, this algorithm can redistribute the transportation loads among a relatively large number of nodes in terms of node degree.

**Fig 8:**
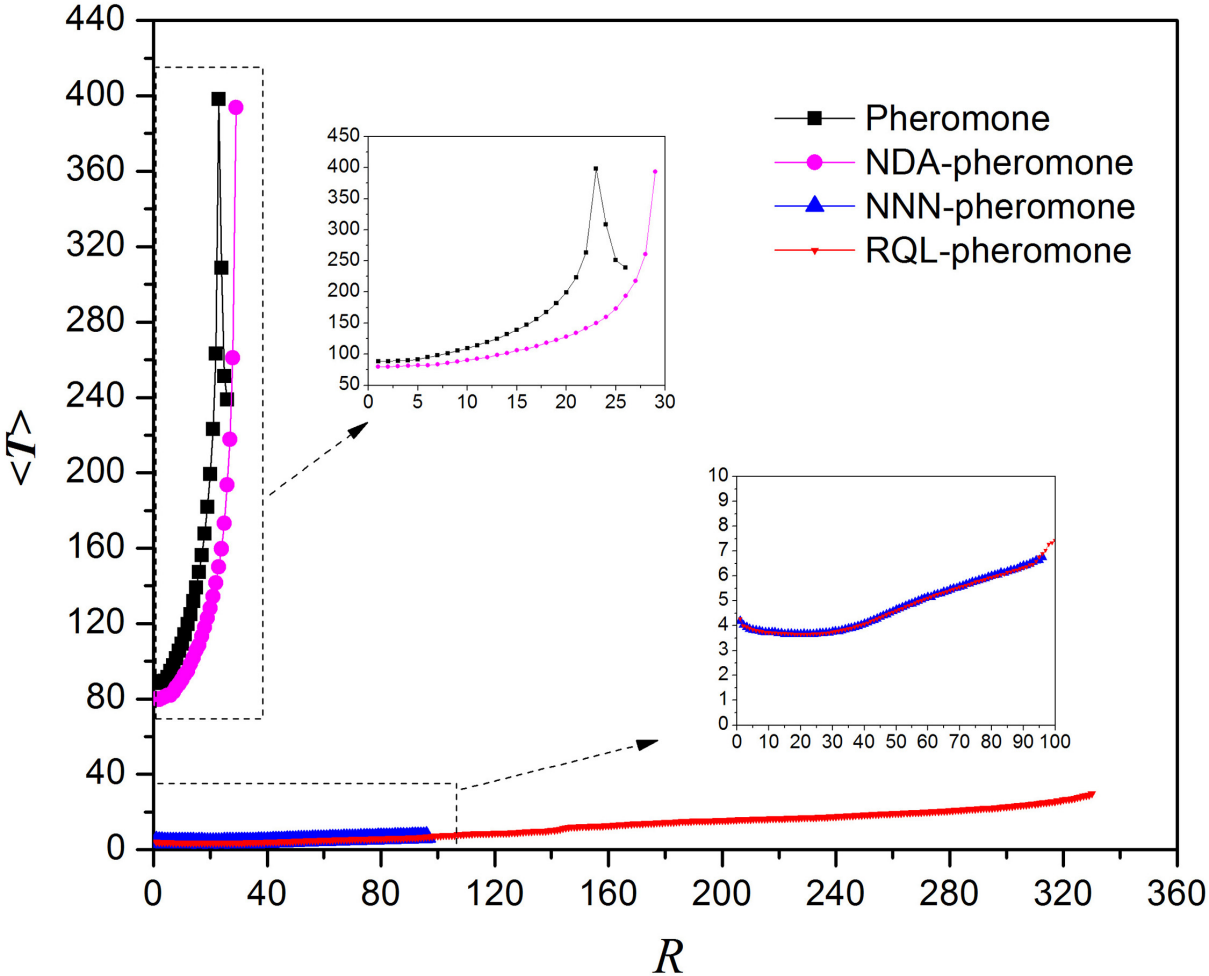
Average transmission time versus *R* for four routing strategies.

The third advantage of restrictive queue length algorithm is that restricting the queue length can save the transmission time, because it can prevent continuous congestion on a node and reduce the waiting time for the queue by forcing information packets to bypass seriously congested nodes until the queue lengths of these nodes fall below the threshold. Fig 8 shows the average transmission time versus *R*. All curves are plotted with *R* ≤ *R*_*c*_, for it could give birth to serious congestion in the network occurs when *R* > *R*_*c*_ and the transmission time would sharply increase. In this figure, the average transmission time for both NDA-local pheromone routing strategy and local pheromone routing strategy increase rapidly. When *R* is close to *R*_*c*_, the average transmission time approaches 400 in both cases. We also find that the rising curve begins to decline as *R* > 23 in the local pheromone routing strategy. This phenomenon is consistent with the fluctuation shown in Fig 2(c). On the contrary, for the NNN-local pheromone routing strategy and the RQL-local pheromone routing strategy, the average transmission time is much less than that of others. When *R* is less than *R*_*c*_ for the NNN-local pheromone routing strategy, the transmission time almost coincides with that of the RQL-local pheromone routing strategy. They are all no more than 10 time steps. More importantly, by restricting the queue length, the average transmission time does not exceed 40 with *R* increasing to *R*_*c*_ = 332.

In summary, the restrictive queue length algorithm is desirable for improving the network capacity and the transmission efficiency.

### Application in other local routing strategies

As mentioned before, there are two other common local routing strategies, namely, local static routing strategy and local dynamic routing strategy. The three proposed algorithms are also applicable to these two local routing strategies. The following results could provide us an answer.

**Fig 9:**
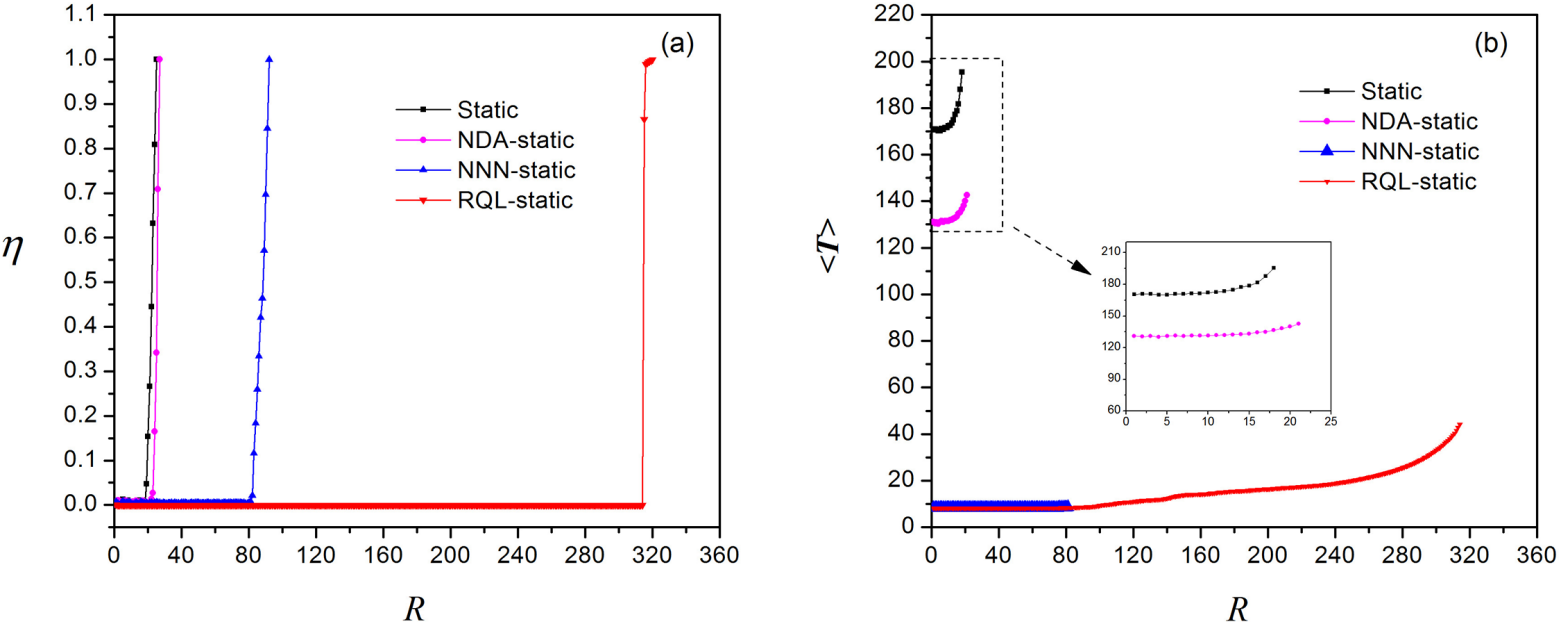
Applying three algorithms to local static routing strategy. (a)The order parameter *η* versus *R*, *η* is normalized; (b)The average transmission time versus *R*.

**Fig 10:**
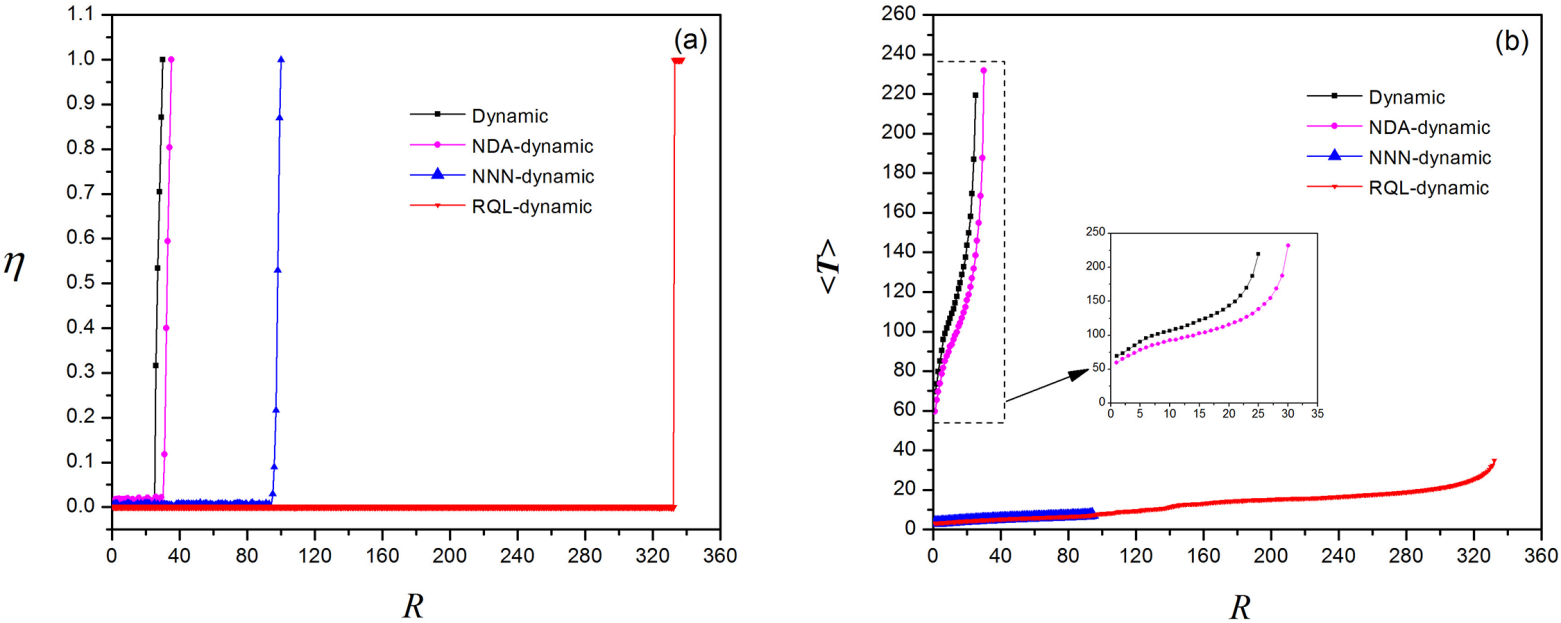
Applying three enhanced algorithms to local dynamic routing strategy. (a)Order parameter *η* versus *R*, *η* is normalized; (b)Average transmission time versus *R*.

**Fig 11:**
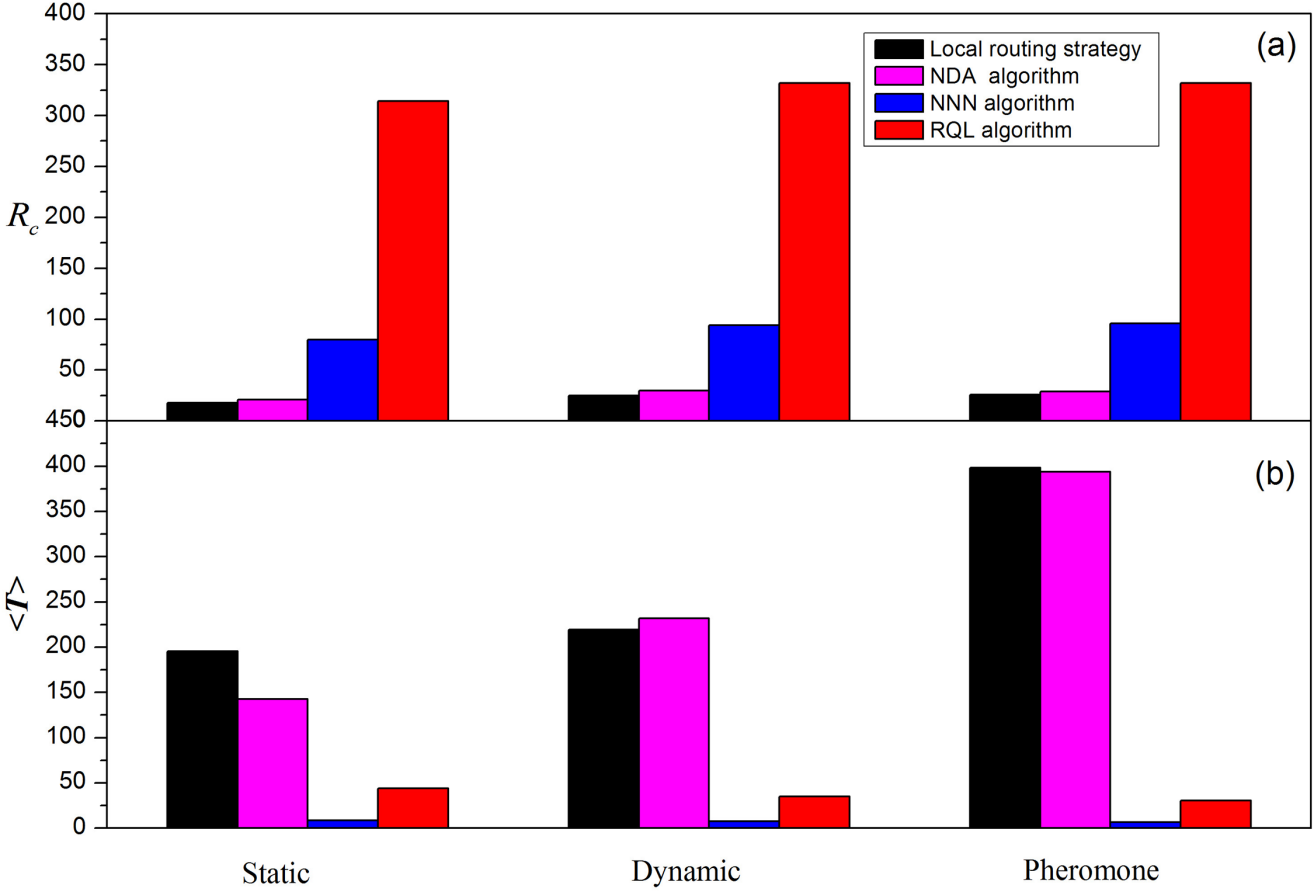
Histograms of *R*_*c*_ and 〈*T*〉 by using local routing strategies and advanced algorithms.

As in the previous simulation, we apply the algorithms to the two local routing strategies and plot the order parameter *η* versus the generation rate of information packets *R* and the average transmission time 〈*T*〉 versus *R*. Fig 9 displays the curves of *η* vs *R* and 〈*T*〉 vs *R* by applying the proposed algorithms to local static routing strategy. The critical generation rate of information packets *R*_*c*_ increases from 18 to 21 after applying the node duplication avoidance algorithm and further rises to 80 by using the next-nearest-neighbor algorithm, finally up to 314 by adopting the restrictive queue length algorithm. The average transmission time 〈*T*〉 decreases from nearly 200 to below 50 after applying these algorithms. Similar results are also obtained by the enhanced local dynamic routing strategy (Fig 10), in which *R*_*c*_ increases from 25 to 332 and 〈*T*〉 decreases from 240 to below 40. In addition, we find that the enhanced value of parameter *β* is between −11 and −7 among which *R*_*c*_ = 340 instead of −3 using local dynamic routing strategy [4]. The contribution of three algorithms for each local routing strategy is demonstrated more clearly in Fig 11.

In general terms, after applying the enhanced algorithm to local routing strategy, *R*_*c*_ rises from below 30 to over 300 and 〈*T*〉 falls from 200–400 to less than 50. These results clearly suggest that the proposed three algorithms are generally applicable to enhance local routing strategies.

### Comparison with global routing strategy

**Fig 12:**
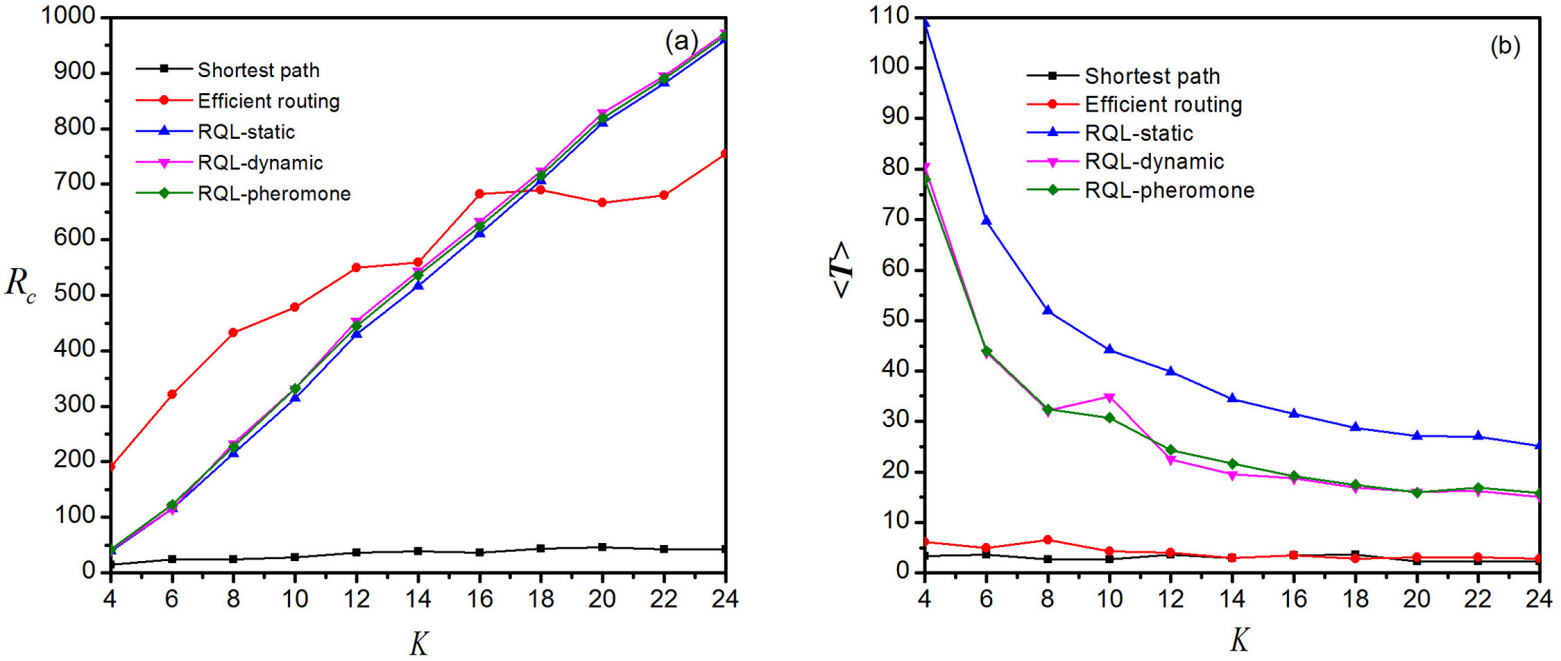
Comparison with global routing protocols. (a) Critical generation rate of information packets *R*_*c*_ versus network average degree *K* for five different routing strategies. (b) Average transmission time versus network average degree.

As illustrated above, global routing strategies are limited to applications in small-scale networks as it is impractical to obtain the real-time information of the whole network. Therefore, it will be a revolutionary leap in both the reduction of computing cost and the improvement of network performance if local routing strategies can perform better than global routing strategies. Fig 12(a) shows the critical generation rate of information packets versus average node degree under five different routing strategies, including the shortest path routing strategy and the efficient routing strategy [9] as well as three local routing strategies improved by restrictive queue length algorithm. It clearly reveals that the value of *R*_*c*_ of the three enhanced local routing strategies increases linearly with the degree and is higher than that of global routing strategies as *K* > 16. With growing *K*, the gap between them is widening. Meanwhile, the average transmission time is below 20 with RQL-local dynamic routing strategy or RQL-local pheromone routing strategy, slightly higher than that by global routing strategy. This means that under certain circumstances the network capacity and transmission efficiency produced by the enhanced local routing strategies are better than that by global routing strategies. These results have great implications for network design and further demonstrates that local routing strategy can be highly effective while keeping the implementation cost low.

## Conclusions

For the sake of improving the efficiency of local routing strategies, three advanced algorithms are proposed in this paper. The first one is the node duplication avoidance algorithm. It could effectively avoid the circulation of packets in the process of transmission. The second one is the next-nearest-neighbor algorithm which is based on the next-nearest-neighbor theory. This algorithm enhances the network performance by enlarging the search space and effectively alleviates the roundabout probabilities. Finally, in order to avoid excessive traffic loads on hub nodes, we propose the restrictive queue length algorithm, which successfully enhances the critical generation rate and the average transmission time. All in all, upon applying the algorithms to local routing strategies, the critical generation rate of information packages increases by over tenfold and the average transmission time reduces by 70–90 percent. Moreover, under certain circumstances, the proposed algorithms for local routing strategies show a higher efficiency than the global routing strategies. This is a revolutionary leap since local routing strategies possess both advantages of lower computing cost and much simpler implementation.
