# Correction: Advanced Algorithms for Local Routing Strategy on Complex Networks

**DOI:** 10.1371/journal.pone.0163075

**Published:** 2016-12-30

**Authors:** Ben-Chuan Lin, Bo-Kui Chen, Ya-Chun Gao, Chi K. Tse, Chuan-Fei Dong, Li-Xin Miao, Bing-Hong Wang

The authors’ names are all spelled incorrectly. The correct spelling for each author is:

Ben-Chuan Lin, Bo-Kui Chen, Ya-Chun Gao, Chi K. Tse, Chuan-Fei Dong, Li-Xin Miao, Bing-Hong Wang

The correct citation is:

Lin B-C, Chen B-K, Gao Y-C, Tse CK, Dong C-F, Miao L-X, et al. (2016) Advanced Algorithms for Local Routing Strategy on Complex Networks. PLoS ONE 11(7): e0156756. doi:10.1371/journal.pone.0156756

There are two errors in the author affiliations:

The affiliation for the second author, Bo-Kui Chen, is incorrect. Bo-Kui Chen is not affiliated with #3 but with #2, School of Computing, National University of Singapore, Singapore, Singapore

The affiliation for the fifth author, Chuan-Fei Dong, is incorrect. Chuan-Fei Dong is not affiliated with #8 but with: Princeton Plasma Physics Laboratory and Department of Astrophysical Sciences, Princeton University, Princeton, New Jersey, United States of America.
